# The Current State of Anterior Cruciate Ligament Reconstruction: Graft Selection, Lateral Augmentation, Rehabilitation, and Return-to-Play

**DOI:** 10.1007/s12178-026-10060-9

**Published:** 2026-10-14

**Authors:** Jeffrey George, Andrew J. Moyal, Isha K. Shah, Robert J. Burkhart, Louis S. Kang, Jacob G. Calcei, James E. Voos

**Affiliations:** 1 Department of Orthopaedic Surgery, University Hospitals Drusinsky Sports Medicine Institute, 3999 Richmond Road, OH 44122 Beachwood, USA; 2 Case Western Reserve University School of Medicine, Cleveland, OH USA

**Keywords:** Anterior cruciate ligament reconstruction, Graft selection, Lateral extra-articular augmentation, Return to play, Psychological readiness, Reinjury

## Abstract

**Purpose of Review:**

Anterior cruciate ligament reconstruction (ACLR) reliably restores anteroposterior stability; however, the rate of return to prior level of play and incidence of recurrent ACL injury have improved little over the past two decades. The purpose of this review is to describe the current state of ACLR with respect to graft selection, lateral extra-articular augmentation, rehabilitation and return to play, including the role of psychological readiness, and the specific rehabilitation protocol utilized by the senior author.

**Recent Findings:**

Graft utilization has shifted substantially. Quadriceps tendon autograft accounted for less than 5% of primary reconstructions prior to 2014 and approximately 20% by 2023. Contemporary registry data demonstrate smaller differences in revision risk between autograft types than previously reported. Randomized and comparative data support lateral extra-articular tenodesis in selected high-risk knees, with the largest effect reported in the revision setting. Return-to-play practice has shifted from fixed timelines toward criteria-based clearance. However, a subset of athletes do not pass a full clearance battery, and some athletes who do pass and observe recommended timeframes still sustain reinjury. Limb symmetry index may overestimate functional recovery, while test batteries and thresholds vary considerably between institutions. Psychological readiness is independently associated with both return to sport and second ACL injury.

**Summary:**

Graft selection should be matched to the patient profile, and lateral augmentation should be reserved for high-risk knees. Return to play should be determined by a combination of time from surgery, functional criteria, and psychological readiness. Future study should focus on validating return-to-play criteria against reinjury endpoints and on standardizing how these criteria are defined and reported.

## Introduction

Anterior cruciate ligament (ACL) injury is among the most common and most consequential injuries sustained by athletes [1, 2]. Analysis of a United States national insurance database identified 931,186 ACL tears between 2010 and 2020 [1]. Incidence is highest among adolescents, and adolescent female athletes sustain ACL injuries at approximately 1.56 times the rate of male athletes, with the greatest per-season risk in soccer, basketball, and gymnastics [2]. Injury rates are higher in club sport than in school-sponsored sport [2].

Anterior cruciate ligament reconstruction (ACLR) is among the most frequently performed procedures in orthopaedic sports medicine and successive technical refinements have been aimed at optimizing outcomes in high-demand athletes [3]. Despite this, functional outcomes have not improved proportionally. Approximately 65% of athletes return to preinjury level of sport and 55% return to competitive sport [4]. Among athletes younger than 25 years who return to pivoting sport, the combined rate of ipsilateral graft rupture and contralateral ACL injury approaches 23% [5]. This discrepancy has led to increasing emphasis on discussion of graft type, incorporation of lateral ligamentous repair/reconstruction, and parameters for return to play clearance.

The purpose of this narrative review is to describe the current state of ACLR with respect to graft selection, lateral augmentation, rehabilitation, and return to play. In doing so, the authors sought to answer the following questions: (1) Which graft should be selected for a given patient profile, and what accounts for the recent increase in quadriceps tendon utilization? (2) Which patients benefit from lateral extra-articular augmentation? (3) Does the senior author’s rehabilitation protocol differ according to graft choice or the addition of LET? (4) Should return to play be governed by time from surgery, by functional criteria, or by both? (5) What is the role of psychological readiness in return-to-play decision-making?

## Graft Selection

### Utilization Trends

Serial survey data from the ACL Study Group spanning 1998 to 2023 document longitudinal changes in graft preference. Over that period, bone–patellar tendon–bone (BTB) autograft use declined gradually, hamstring tendon (HT) autograft use rose and subsequently fell, allograft use remained low among surveyed surgeons, and quadriceps tendon (QT) autograft use rose sharply [6]. QT accounted for no more than 5% of primary reconstructions between 1998 and 2014 but approached 20% by 2023 [6]. In the revision setting, hamstring use declined abruptly after 2016, with concurrent increases in both BTB and QT utilization [6].

### Bone–Patellar Tendon–Bone

BTB autograft remains the reference standard for the young, high-demand pivoting athlete. Its advantages are both mechanical and biological: bone-to-bone incorporation at both tunnels, predictable and rigid fixation, and an extensive track record in competitive populations [3]. A recent meta-analysis restricted to patients younger than 25 years found graft failure rates of 11.1% for HT, 5.1% for BTB, and 2.5% for QT. Failure was significantly higher with HT than with BTB, whereas QT did not differ significantly from either graft [7].

The trade-off is donor-site morbidity. Anterior knee pain and pain with kneeling remain the most frequent complaints, and patellar fracture and patellar tendon rupture are rare but consequential [3]. These considerations are particularly relevant in athletes whose sport involves frequent kneeling or direct patellar contact. Graft characteristics and typical indications are summarized in Table 1.

**Table 1:** Autograft and allograft options for anterior cruciate ligament reconstruction

Graft	Advantages	Disadvantages	Typical indication
Bone–patellar tendon–bone	Bone-to-bone healing at both tunnels; rigid fixation; extensive track record in competitive athletes	Anterior knee and kneeling pain; rare patellar fracture or patellar tendon rupture	Young, high-demand pivoting athlete; reference standard
Quadriceps tendon	Large cross-sectional area; all–soft tissue or bone-block configuration; lower harvest-site pain than BTB (0–11.5%)	Outcomes appear surgeon-volume dependent; extension strength recovery pattern differs from hamstring graft	Primary and revision reconstruction; attractive where kneeling demands are high
Hamstring tendon	Low harvest-site pain; technically familiar; registry revision rates comparable to other autografts	Residual flexion weakness; tunnel widening; historical concern in young female athletes	Selected primary reconstruction; declining use in North American practice
Allograft	No donor-site morbidity; shorter operative time; useful when autograft supply is limited	Substantially higher failure rate in patients under 25 returning to pivoting sport	Older or lower-demand patients; multiligament injury; selected revisions

*BTB* bone–patellar tendon–bone, *HT* hamstring tendon, *QT* quadriceps tendon

### Hamstring Tendon

Preference for hamstring autograft has declined among surveyed ACL Study Group surgeons, while a recent meta-analysis found higher failure rates with HT than BTB in patients younger than 25 years [6, 7]. Residual knee flexion weakness and tunnel widening are recognized limitations [3, 8]. Contemporary registry data, however, temper the concerns regarding revision risks. In a Swedish Knee Ligament Registry analysis of 44,513 patients, two-year revision rates were 2.2% for QT, 2.5% for BTB, and 2.3% for HT, while graft type was not associated with revision hazard in adjusted analysis [9]. A separate cohort of 27,715 patients undergoing ACLR between 2012 and 2023 found that QT was associated with lower revision risk than HT only in females younger than 22 years [10].

### Quadriceps Tendon

QT autograft has been the principal beneficiary of the shift away from hamstring graft. Advantages include a large and reliably obtainable cross-sectional area, the option of an all–soft tissue or bone-block configuration, lower donor-site morbidity than BTB, and donor graft-site pain reported in 0% to 11.5% of patients across studies [3, 11, 12]. A systematic review of randomized trials has demonstrated comparable graft failure, laxity, complication rates, and patient-reported outcomes across QT, HT, and BTB [12].

Two caveats warrant emphasis. First, graft-specific strength recovery is not neutral. Recent synthesis of randomized data indicates that hamstring autografts favor recovery of knee extension strength while quadriceps autografts favor recovery of flexion strength, with direct implications for rehabilitation programming and interpretation of strength-based clearance criteria [13]. Second, QT outcomes appear sensitive to surgeon volume. Danish registry data demonstrated a QT revision rate of 6.4% at low-volume centers compared with 2.9% at high-volume centers, with an adjusted revision hazard ratio of 2.3 for low-volume practice [14].

The current state of QT literature is continuing to grow, with increasing evidence supporting its use as a viable graft, especially when an all soft-tissue graft is needed. The aggregate signal is that QT performs comparably to established autografts in experienced hands [9–12]. The volume-dependence finding is the more actionable of the two [14]. A surgeon adopting QT should anticipate a learning curve and should not assume that outcomes published from high-volume centers apply to early experience.

### Allograft

Allograft should be avoided in young athletes. Comparative data demonstrate substantially higher failure rates with allograft in patients 19 years or younger, with pooled failure rates of 25.5% for allograft, 16.6% for HT autograft, and 8.5% for BTB autograft [15]. More recent propensity-matched analysis in adolescents has suggested the difference narrows after adjustment for baseline characteristics, but the weight of evidence continues to favor autograft in adolescent patients returning to sport [15]. Allograft retains a defensible role in older or lower-demand patients, in multiligament reconstruction where autograft supply is limited, and in selected revision scenarios [3].

## Lateral Extra-Articular Augmentation

Isolated intra-articular reconstruction may not fully restore anterolateral rotatory stability within high-risk patients. Persistent pivot shift after appropriately performed ACLR has been associated with worse patient-reported outcomes and graft failure, driving renewed interest in lateral extra-articular tenodesis (LET) and anterolateral ligament (ALL) reconstruction [16].

The STABILITY randomized clinical trial provides the strongest primary evidence. The trial enrolled 618 patients aged between 14 and 25 years with at least two of three risk features (grade 2 or greater pivot shift, intention to return to high-risk pivoting sport, and generalized ligamentous laxity) and randomized patients to hamstring autograft ACLR with or without a modified Lemaire LET [17]. At two-year follow-up, the addition of LET significantly reduced graft rupture and similarly the clinical-failure endpoint of graft rupture or persistent rotatory laxity [17]. Though LET did not significantly increase overall rates of minor medical events, minor surgical events, or reoperation, hardware irritation and removal were more frequent [18].

The revision setting shows an even larger effect. A retrospective comparative series with a mean follow-up beyond six years reported graft failure in 3.3% of revision ACLR performed with LET compared with 18.3% of isolated revision ACLR [19]. A systematic review and meta-analysis of comparative studies in revision ACLR similarly found that the addition of LET or ALL reconstruction improved subjective IKDC scores, restored rotational stability, and reduced failure rates relative to isolated revision reconstruction [20]. A large administrative database analysis found LET was correlated with a reduced risk of subsequent revision ACLR [21].

Contemporary indications for LET, reflected in both published series and current practice patterns, include revision reconstruction, high-grade pivot shift, generalized ligamentous laxity or knee hyperextension, and the young athlete returning to pivoting or contact sport [16, 22]. Technique selection, including modified Lemaire, MacIntosh, and anatomic ALL reconstruction, remains an area of debate and has recently been reviewed in detail [16].

The authors perform lateral augmentation routinely in revision reconstruction, consistent with the magnitude of benefit reported during revision ACLR, and selectively in primary reconstruction when two or more stability risk features are present [17]. Importantly, LET is not a substitute for appropriately performed primary reconstruction. Attention to tunnel position, graft selection, and treatment of concomitant meniscal and slope-related pathology remains essential.

## Return to Play: Timing and Criteria

### The Case for Time-Based Clearance

Temporal evidence is among the most robust in this literature. In the Delaware–Oslo ACL cohort, each additional month of delayed return to sport up to nine months was associated with an approximately 51% reduction in reinjury risk [23]. Application of time-based and functional return-to-sport criteria was associated with an estimated 84% lower reinjury risk, although the comparison did not reach statistical significance [23]. In a separate cohort, young athletes returning before nine months sustained secondary ACL injuries at approximately seven times the rate of athletes returning greater than nine months following ACLR [24]. A meta-analysis found that patients who sustained a second ACL injury returned to sport 25 days earlier (95% CI, 9.5–40.4 days) than those who did not; however, this trend was not seen in professional athletes [25]. Subsequent work from the Delaware–Oslo cohort has further suggested that activity level and functional readiness, rather than chronological age itself, are the critical determinants [26]. Graft ligamentization may continue for twelve to twenty-four months and varies considerably between individuals [27].

### The Case for Criteria-Based Clearance

Criteria-based clearance rests on the premise that measurable functional recovery, not elapsed time, should govern the decision. The most frequently cited evidence comes from a cohort of 158 male professional athletes, of whom 26 (16.5%) sustained graft rupture at a mean of 105 days after return to sport [28]. Failure to meet all six discharge criteria before returning to team training was associated with a hazard ratio of 4.1 for graft rupture [28]. A typical contemporary battery includes isokinetic quadriceps and hamstring strength testing, a hop test battery, assessment of movement quality, and increasingly a patient-reported measure of psychological readiness when determining appropriate return to sport [28, 29]. These domains and their supporting evidence are summarized in Table 2.

**Table 2:** Common return-to-play criteria and evidentiary considerations

Domain	Representative test	Common threshold	Evidentiary considerations
Time from surgery	Postoperative interval	≥ 9 months	Each month of delay to 9 months associated with ~ 51% reinjury reduction; return before 9 months associated with ~ 7-fold higher new injury rate
Quadriceps strength	Isokinetic testing at 60°/s	LSI ≥ 90%	LSI may overestimate recovery; estimated preinjury capacity more sensitive for predicting second injury
Functional capacity	Single, triple, and crossover hop battery	LSI ≥ 90% each test	Failure to meet full discharge battery associated with HR 4.1 for graft rupture; passing has not been shown to reduce second injury risk
Movement quality	Drop vertical jump; qualitative screening	No validated standard	Marked heterogeneity between institutions; limits cross-cohort comparison
Psychological readiness	ACL-RSI	Commonly ≥ 65	Independently associated with return to sport and with second ACL injury; scores plateau early after reconstruction

*ACL-RSI* anterior cruciate ligament–return to sport after injury, *HR* hazard ratio, *LSI* limb symmetry index

### Remaining Questions in Return-to-Play Assessment

Criteria-based clearance represents the best available synthesis of the return-to-play literature and has meaningfully advanced practice [29]. Given the widespread adoption of these criteria, their remaining limitations warrant examination.

First, a subset of athletes do not pass a full clearance battery, while athletes who do pass criteria while observing recommended timeframes still sustain reinjury [30]. A systematic review with meta-analysis reported that 42.7% of patients passed return-to-sport criteria and that 14.4% of those who passed sustained a second ACL injury, with no statistically significant association between passing criteria and reduced second injury risk [31]. The authors of that review noted that the quality of available evidence precludes a definitive conclusion. This finding should not be interpreted as an argument against criteria-based clearance, but rather as an open question regarding what current batteries measure and which residual factors they fail to capture.

Second, limb symmetry index (LSI) may overestimate functional recovery, as the uninvolved limb also deconditions during rehabilitation [30]. When preoperative uninvolved-limb performance is used as the reference standard, the difference is substantial. In one cohort, 57.1% of patients achieved 90% LSI across quadriceps strength and all hop tests, whereas only 28.6% met the same 90% threshold against estimated preinjury capacity, and estimated preinjury capacity was more sensitive than LSI in predicting second ACL injury [30]. This offers one explanation for reinjury among athletes who have met symmetry thresholds, and supports preinjury or estimated preinjury referencing where baseline testing is feasible.

Third, batteries, thresholds, and integration of patient-reported outcome measures vary considerably between institutions. Systematic and scoping reviews have documented marked heterogeneity and a lack of standardization in how return to sport is assessed and reported [29, 32]. This limits comparison across published cohorts and may account for variability in reinjury rates reported between centers [32]. Standardization of reporting would meaningfully improve comparability of future studies.

### Psychological Readiness

Kinesiophobia and fear of reinjury are commonly seen following ACLR and have been shown to impede return to sport, alter measurable knee biomechanics, and potentially increase the risk of re-rupture [33]. Although current clinical practice guidelines recognize the role of psychological factors in rehabilitation, patient-reported outcome measures and psychological readiness are infrequently incorporated into rehabilitation protocols [33]. This may contribute in part to the discrepancy between high rates of successful surgical reconstruction and considerably lower rates of return to competitive play [4].

The ACL-Return to Sport after Injury (ACL-RSI) scale is the most widely used instrument in this domain, assessing emotion, confidence in performance, and risk appraisal. The predictive value of ACL-RSI extends beyond return to sport alone. Among 329 patients who returned to sport, 52 (16%) sustained a second ACL injury, and lower psychological readiness plus younger age were identified as risk factors [34]. Importantly, the relationship between psychological readiness and reinjury may vary by assessment timing, with higher psychological readiness at three months postoperatively associated with a higher incidence of second ACL injury [35].

The trajectory of these scores affects how the instrument should be used. A systematic review with meta-analysis of ACL-RSI scores over time demonstrates that psychological readiness improves early after reconstruction, with comparatively little subsequent improvement until two years or beyond [36]. A single low score at the point of clearance may therefore reflect a stable feature of that athlete’s recovery rather than a transient state. This supports serial measurement and early intervention rather than a single assessment at the end of rehabilitation [36].

Interest has accordingly shifted toward psychologically informed rehabilitation, graded exposure to sport-specific movements, and targeted interventions such as sports-specific visualization, knee bracing, and kinesiotaping [33]. Network meta-analysis has supported integration of psychological and physical assessment for optimizing return-to-sport outcomes [37]. The authors administer the ACL-RSI as part of the clearance battery (Table 2) and regard lower scores as an indication for further targeted rehabilitation rather than for clearance with reassurance alone [33, 37].

### Key Principles

**Fig. 1:**
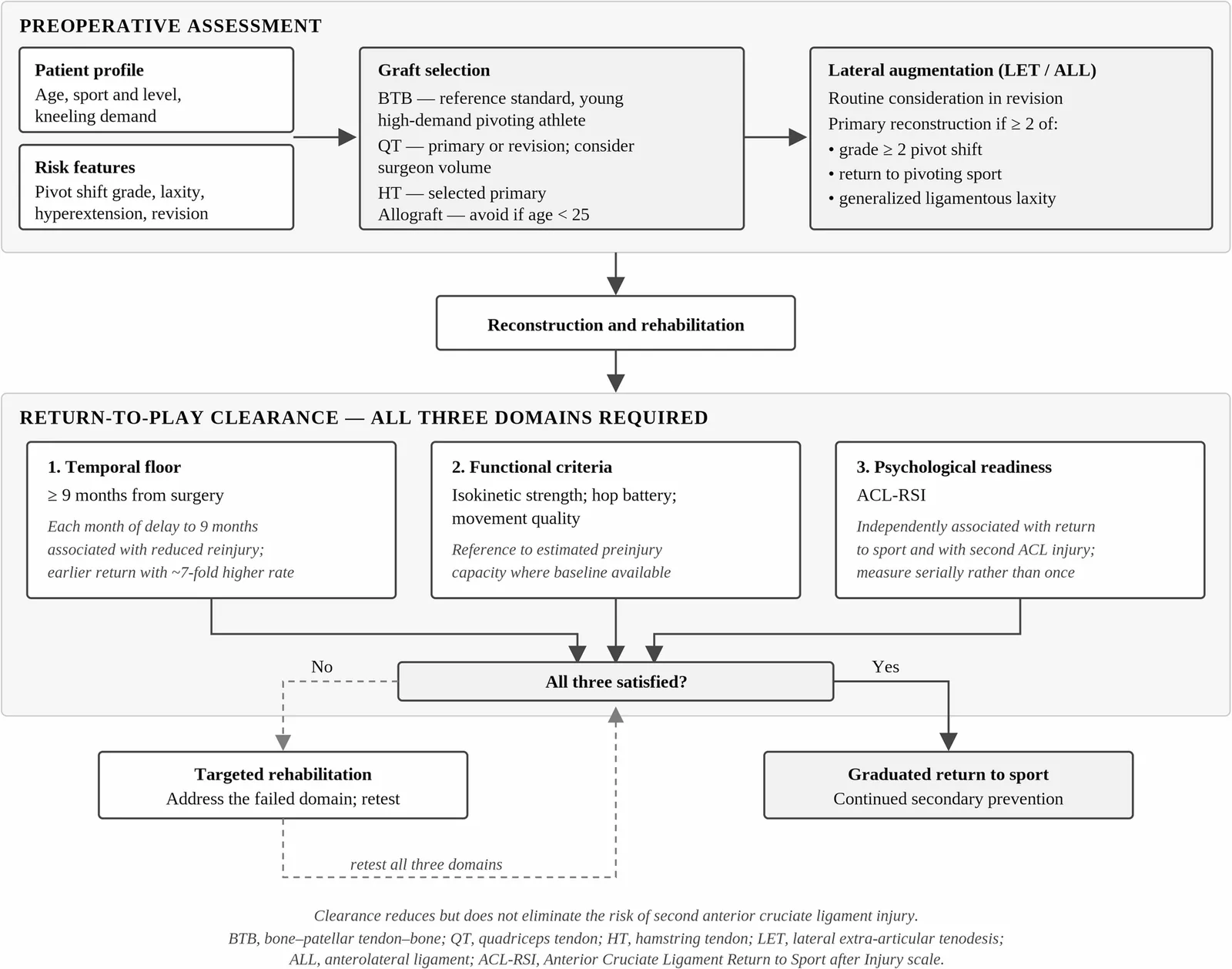
Proposed framework for decision-making in anterior cruciate ligament reconstruction. Preoperative assessment of patient age, sport demand, pivot shift grade, and ligamentous laxity informs graft selection and the decision regarding lateral extra-articular augmentation. Postoperative clearance requires concurrent satisfaction of three domains: a temporal floor of approximately nine months, passage of a functional criteria battery referenced where possible to estimated preinjury capacity, and formal assessment of psychological readiness. Failure in any single domain returns the athlete to targeted rehabilitation rather than to sport

Given the above, several key principles can be applied to return-to-play decision-making following ACLR (Fig. 1):

1. A temporal floor of approximately nine months should be observed. Each additional month of delay to nine months is associated with reduced reinjury risk, and return prior to nine months is associated with a substantially higher rate of new injury [23, 24].
2. Functional criteria should be assessed formally rather than assumed, as failure to meet a full discharge battery is associated with a fourfold increase in graft rupture [28, 29]. Where preoperative baseline testing of the uninvolved limb is available, estimated preinjury capacity is preferred to limb symmetry index as the reference standard [30].
3. Psychological readiness should be measured as a discrete domain rather than inferred from physical performance, and should be assessed serially rather than once at the point of clearance [33, 34, 36].
4. Athletes and families should be counseled that clearance may reduce but does not eliminate the risk of recurrent ACL injury [23, 28, 31]. This counseling is particularly important in athletes younger than 25 years returning to pivoting sport [5].

### Our Protocol

The senior author implements the aforementioned principles in the institution-wide protocol for ACLR rehabilitation. Regardless of graft choice, rehabilitation progression consists of time- and performance-based metrics. As ligamentization occurs within a specific timeframe, the 8–9-month time point is utilized as a baseline for clearance, along with performance metrics [23–25, 27, 28]. The senior author’s protocol is not independently modified by the addition of LET.

Patients will partake in closed- and open-kinetic-chain work for three months, with consistent and heavy use of occlusion training for quadriceps strength and fan-bike cycling for cardiovascular endurance and quadriceps strength. At three months, patients transition to turf work with straight-line activities, including jogging, with a goal of building back cardiovascular endurance. At six months, patients begin cutting and pivoting activities, along with limb-symmetry index testing to track progression of quadriceps and hamstring strength. Despite the initiation of functional testing, patients are not cleared until the 8–9-month time point. Patients will continue with their rehabilitation until they reach 80–90% LSI on functional and strength-based testing. Once greater than 90% is achieved on LSIs, patients will be cleared for return to sport [23, 28, 29]. If needed, we will continue to assess progression through the 12-month time point.

Along with this rehabilitation protocol, the senior author emphasizes a slow and gradual increase in sport-specific volume, with multiple weeks of practice or simulation prior to competitive play. In patients undergoing more complex procedures, including multiligament reconstruction or revision ACLR, a similar protocol is used with a slower progression.

## Conclusions

Contemporary ACLR is best understood as three linked decisions rather than a single operation. Graft selection should be matched to patient profile rather than applied uniformly (Table 1). BTB remains the reference standard in the young high-demand athlete. QT performs comparably in experienced hands with the caveat that outcomes appear volume-dependent. Hamstring autograft performs better in registry data than current North American practice patterns would suggest. Allograft should be avoided in young pivoting athletes. Lateral extra-articular augmentation is now an evidence-supported adjunct, with the largest benefit reported in revision reconstruction and in primary reconstruction of the high-risk knee. Return to play should be governed by a combination of time from surgery, functional criteria, and psychological readiness, none of which is sufficient alone (Table 2; Fig. 1).

Future study should focus on validation of return-to-play criteria against reinjury endpoints, as the batteries in widest use have not been shown to reduce second injury; standardization of how these criteria are defined and reported, in order to allow comparison between cohorts; continued registry surveillance of quadriceps tendon autograft as adoption broadens beyond high-volume centers; and completion of ongoing randomized work extending lateral augmentation to patellar and quadriceps tendon grafts.

## Data Availability

No datasets were generated or analysed during the current study.

## Key References

- Getgood AMJ, Bryant DM, Litchfield R, Heard M, McCormack RG, Rezansoff A, et al. Lateral extra-articular tenodesis reduces failure of hamstring tendon autograft anterior cruciate ligament reconstruction: 2-year outcomes from the STABILITY study randomized clinical trial. Am J Sports Med. 2020;48(2):285–97.
  - ○* The largest multicenter randomized trial of lateral extra-articular tenodesis in high-risk primary reconstruction and a major basis for current indications.*
- Grindem H, Snyder-Mackler L, Moksnes H, Engebretsen L, Risberg MA. Simple decision rules can reduce reinjury risk by 84% after ACL reconstruction: the Delaware-Oslo ACL cohort study. Br J Sports Med. 2016;50(13):804–8.
  - ○ *Identifies the dose-response association between delayed return to sport and reduced reinjury that underpins the nine-month temporal floor.*
- Kyritsis P, Bahr R, Landreau P, Miladi R, Witvrouw E. Likelihood of ACL graft rupture: not meeting six clinical discharge criteria before return to sport is associated with a four times greater risk of rupture. Br J Sports Med. 2016;50(15):946–51.
  - ○* Widely cited evidence that failure to meet functional discharge criteria is associated with graft rupture in professional athletes.*
- Losciale JM, Zdeb RM, Ledbetter L, Reiman MP, Sell TC. The association between passing return-to-sport criteria and second anterior cruciate ligament injury risk: a systematic review with meta-analysis. J Orthop Sports Phys Ther. 2019;49(2):43–54.
  - ○* Found no statistically significant association between passing current criteria and reduced second injury risk, defining a central unresolved question in return-to-play assessment. *
- Wellsandt E, Failla MJ, Snyder-Mackler L. Limb symmetry indexes can overestimate knee function after anterior cruciate ligament injury. J Orthop Sports Phys Ther. 2017;47(5):334–8.
  - ○* Shows that symmetry-based thresholds may overestimate knee function and permit premature clearance. *
- McPherson AL, Feller JA, Hewett TE, Webster KE. Psychological readiness to return to sport is associated with second anterior cruciate ligament injuries. Am J Sports Med. 2019;47(4):857–62.
  - ○* Links lower psychological readiness in younger patients to subsequent injury, supporting its inclusion in clearance batteries. *
- Nedder VJ, Raju AG, Moyal AJ, Calcei JG, Voos JE. Impact of psychological factors on rehabilitation after anterior cruciate ligament reconstruction: a systematic review. Sports Health. 2025;17(2):291–8.
  - ○* Summarizes the psychological factors affecting rehabilitation after ACLR and the gap between current clinical practice guidelines and routine incorporation of psychological measures.*

## References

[1] Bergstein VE, Ahiarakwe U, Haft M, Fox H, Best MJ. Decreasing Incidence of Anterior Cruciate Ligament Tears and Increasing Utilization of Anterior Cruciate Ligament Reconstruction in the United States From 2010 to 2020. Arthroscopy. 2025;41(6):1912. 8.e1. 10.1016/j.arthro.2024.08.018 39214428

[2] Childers J, Eng E, Lack B, Lin S, Knapik DM, Kaplan DJ, et al. Reported Anterior Cruciate Ligament Injury Incidence in Adolescent Athletes Is Greatest in Female Soccer Players and Athletes Participating in Club Sports: A Systematic Review and Meta-analysis. Arthroscopy. 2025;41(3):774–e842. 10.1016/j.arthro.2024.03.050. 38692337

[3] Homan MD, Braaten JA, Banovetz MT, Monson JK, Kennedy NI, LaPrade RF. Principles for Optimizing Anterior Cruciate Ligament Reconstruction Outcomes in Elite Athletes: A Review of Current Techniques. Annals Joint. 2024;9:19. 10.21037/aoj-22-40. PMC11061659 38694814

[4] Ardern CL, Taylor NF, Feller JA, Webster KE. Fifty-five Per Cent Return to Competitive Sport Following Anterior Cruciate Ligament Reconstruction Surgery: An Updated Systematic Review and Meta-analysis Including Aspects of Physical Functioning and Contextual Factors. Br J Sports Med. 2014;48(21):1543–52. 10.1136/bjsports-2013-093398. 25157180

[5] Wiggins AJ, Grandhi RK, Schneider DK, Stanfield D, Webster KE, Myer GD. Risk of Secondary Injury in Younger Athletes After Anterior Cruciate Ligament Reconstruction: A Systematic Review and Meta-analysis. Am J Sports Med. 2016;44(7):1861–76. 10.1177/0363546515621554. PMC5501245 26772611

[6] Schroeder GG, Hollyer I, Raji Y, Akhtar M, Kaeding CC, Spalding T, et al. 25-Year Trends in Anterior Cruciate Ligament Reconstruction: Results From the Biennial ACL Study Group Survey From 1998 to 2023. J ISAKOS. 2026;16:101039. 10.1016/j.jisako.2025.101039. 41274559

[7] Petit CB, Hussain ZB, Read PJ, McPherson AL, Pradip K, Petushek EJ, et al. Graft Failure Rates in Bone-Patellar Tendon-Bone, Hamstring, and Quadriceps Tendon Autografts in Patients Younger Than 25 Years: A Meta-analysis. Am J Sports Med. 2026;54(9):2300–12. 10.1177/03635465261415842. 41733021

[8] Hali NZ, Punjani H, Tahir M, Ng J, Aweid O, Boutefnouchet T, et al. No Difference in Functional Outcomes and Stability but Lesser Tunnel Widening With Tibial Suspensory Fixation Compared With Interference Screw in Hamstring Autograft Anterior Cruciate Ligament Reconstruction: A Systematic Review. Arthroscopy. 2026. 10.1002/arj.70360. 42329336

[9] Rizvanovic D, Waldén M, Forssblad M, Cristiani R, von Essen C, Stålman A. Patient-Reported Outcomes and Revision Rates After ACL Reconstruction With Quadriceps Versus Hamstring and Patellar Tendon Autografts: Sex-Stratified Results From the Swedish Knee Ligament Registry. Am J Sports Med. 2026;54(3):558–68. 10.1177/03635465251404876. PMC12916877 41588802

[10] Lehman CR, Prentice HA, Reyes C, Mansfield C, Yang JS, Maletis GB. Risk of Revision and Reoperation After ACL Reconstruction: Comparison of Quadriceps Tendon, Patellar Tendon, and Hamstring Autografts Stratified by Patient Sex and Age: A Cohort Study of 27,715 Patients From 2012 to 2023. Am J Sports Med. 2026;54(5):1011–26. 10.1177/03635465261428123. 41846566

[11] Hopper HM, Harris M, Wyatt P, Cyrus J, Smith M, O’Neill CN, et al. Quadriceps Tendon Autograft Produces Favorable Return to Sport, Strength, Range of Motion, Retear Rate, and Midterm Outcomes After Anterior Cruciate Ligament Reconstruction: A Systematic Review. Arthroscopy. 2026;42(2):539–58. 10.1002/arj.70092. 41879151

[12] White T, Castro M, Antonio L, Hing W, Tudor F, Sattler L, Quadriceps. Hamstring and Patella Tendon Autografts for Primary Anterior Cruciate Ligament Reconstruction Demonstrate Similar Clinical Outcomes, Including Graft Failure, Joint Laxity and Complications: A Systematic Review With Meta-analysis of Randomised Controlled Trials. Knee Surgery, Sports Traumatology. Arthroscopy. 2026;34(5):1631–46. 10.1002/ksa.12755. PMC13122740 40679231

[13] Heinz M, Lettner J, Topkarci YB, Królikowska A, Ramadanov N, Ostojic M, et al. Hamstring Autografts Favour Knee Extension Strength Recovery While Quadriceps Autografts Optimise Flexion Strength Recovery: A Systematic Review of Randomised Controlled Trials. J Experimental Orthop. 2026;13(1):e70665. 10.1002/jeo2.70665. PMC12914483 41717284

[14] Lind M, Strauss MJ, Nielsen T, Engebretsen L. Low Surgical Routine Increases Revision Rates After Quadriceps Tendon Autograft for Anterior Cruciate Ligament Reconstruction: Results From the Danish Knee Ligament Reconstruction Registry. Knee Surg Sports Traumatol Arthrosc. 2021;29(6):1880–6. 10.1007/s00167-020-06220-0. 32886156

[15] Cruz AI Jr., Beck JJ, Ellington MD, Mayer SW, Pennock AT, Stinson ZS, et al. Failure Rates of Autograft and Allograft ACL Reconstruction in Patients 19 Years of Age and Younger: A Systematic Review and Meta-analysis. JBJS Open Access. 2020;5(4):e2000106. 10.2106/JBJS.OA.20.00106. PMC8312832 34322650

[16] Veizi E, Oliver-Welsh L, Koutserimpas C, Imat E, Getgood A. Extra-articular Tenodesis for ACL Reconstruction: Who Needs It and Is There a Superior Technique? Curr Rev Musculoskelet Med. 2026;19(1):31. 10.1007/s12178-026-10028-9. PMC13043991 41922887

[17] Getgood AMJ, Bryant DM, Litchfield R, Heard M, McCormack RG, Rezansoff A, et al. Lateral Extra-articular Tenodesis Reduces Failure of Hamstring Tendon Autograft Anterior Cruciate Ligament Reconstruction: 2-Year Outcomes From the STABILITY Study Randomized Clinical Trial. Am J Sports Med. 2020;48(2):285–97. 10.1177/0363546519896333. 31940222

[18] Heard M, Marmura H, Bryant DM, Litchfield R, McCormack RG, MacDonald PB, et al. No Increase in Adverse Events With Lateral Extra-articular Tenodesis Augmentation of Anterior Cruciate Ligament Reconstruction: Results From the STABILITY Randomized Trial. J ISAKOS. 2023;8(4):246–54. 10.1016/j.jisako.2022.12.001. 36646169

[19] Gonzalez W, Isla J, Sandoval A, Figueroa F, Itriago M, Figueroa D. Lateral extra-articular tenodesis reduces graft failure without affecting functional outcomes after revision anterior cruciate ligament reconstruction: A retrospective comparative study with 6-year follow-up. J ISAKOS. 2026;16:101048. 10.1016/j.jisako.2025.101048. 41319872

[20] Boksh K, Sheikh N, Chong HH, Ghosh A, Aujla R. The Role of Anterolateral Ligament Reconstruction or Lateral Extra-articular Tenodesis for Revision Anterior Cruciate Ligament Reconstruction: A Systematic Review and Meta-analysis of Comparative Clinical Studies. Am J Sports Med. 2024;52(1):269–85. 10.1177/03635465231157377. 36960926

[21] Serna J, Kucirek N, Terada-Herzer K, Lansdown D, Ma CB, Zhang AL. Anterior Cruciate Ligament Reconstruction With Lateral Extra-articular Tenodesis Is Associated With Reduced Risk for Revision Anterior Cruciate Ligament Reconstruction in an Insurance Claims Database. Arthroscopy. 2025;41(9):3425–e341. 10.1016/j.arthro.2025.02.015. 40010521

[22] Bartkoski M, Coda R, Mar D, Herda AA, Bernard C, Morey T, et al. Current Trends and Indications for Extra-Articular Reconstruction in Revision ACL Reconstruction: A Cross-Sectional Study. Orthop J Sports Med. 2025;13(11):23259671251386878. 10.1177/23259671251386878. PMC12612503 41244226

[23] Grindem H, Snyder-Mackler L, Moksnes H, Engebretsen L, Risberg MA. Simple Decision Rules Can Reduce Reinjury Risk by 84% After ACL Reconstruction: The Delaware-Oslo ACL Cohort Study. Br J Sports Med. 2016;50(13):804–8. 10.1136/bjsports-2016-096031. PMC4912389 27162233

[24] Beischer S, Gustavsson L, Senorski EH, Karlsson J, Thomee C, Samuelsson K, et al. Young Athletes Who Return to Sport Before 9 Months After Anterior Cruciate Ligament Reconstruction Have a Rate of New Injury 7 Times That of Those Who Delay Return. J Orthop Sports Phys Ther. 2020;50(2):83–90. 10.2519/jospt.2020.9071. 32005095

[25] Piussi R, Simonson R, Zsidai B, Grassi A, Karlsson J, Della Villa F, et al. Better Safe Than Sorry? A Systematic Review with Meta-analysis on Time to Return to Sport After ACL Reconstruction as a Risk Factor for Second ACL Injury. J Orthop Sports Phys Ther. 2024;54(3):161–75. 10.2519/jospt.2023.11977. 38032099

[26] Grindem H, Engebretsen L, Axe MJ, Snyder-Mackler L, Risberg MA, Activity, Readiness F. Not Age, Are the Critical Factors for Second Anterior Cruciate Ligament Injury-The Delaware-Oslo ACL Cohort Study. Br J Sports Med. 2020;54(18):1099–102. 10.1136/bjsports-2019-100623. PMC7442843 32046955

[27] Claes S, Verdonk P, Forsyth R, Bellemans J. The Ligamentization Process in Anterior Cruciate Ligament Reconstruction: What Happens to the Human Graft? A Systematic Review of the Literature. Am J Sports Med. 2011;39(11):2476–83. 10.1177/0363546511402662. 21515806

[28] Kyritsis P, Bahr R, Landreau P, Miladi R, Witvrouw E. Likelihood of ACL Graft Rupture: Not Meeting Six Clinical Discharge Criteria Before Return to Sport Is Associated With a Four Times Greater Risk of Rupture. Br J Sports Med. 2016;50(15):946–51. 10.1136/bjsports-2015-095908. 27215935

[29] Wright A, Reid D, Potts G. Return to Sport (RTS) Tests and Criteria Following an Anterior Cruciate Ligament (ACL) Reconstruction (ACLR): A Scoping Review. Knee. 2025;57:179–99. 10.1016/j.knee.2025.08.010. 40865179

[30] Wellsandt E, Failla MJ, Snyder-Mackler L. Limb Symmetry Indexes Can Overestimate Knee Function After Anterior Cruciate Ligament Injury. J Orthop Sports Phys Therapy. 2017;47(5):334–8. 10.2519/jospt.2017.7285. PMC5483854 28355978

[31] Losciale JM, Zdeb RM, Ledbetter L, Reiman MP, Sell TC. The Association Between Passing Return-to-Sport Criteria and Second Anterior Cruciate Ligament Injury Risk: A Systematic Review With Meta-analysis. J Orthop Sports Phys Therapy. 2019;49(2):43–54. 10.2519/jospt.2019.8190. 30501385

[32] Marom N, Xiang W, Wolfe I, Jivanelli B, Williams RJ, Marx RG. High Variability and Lack of Standardization in the Evaluation of Return to Sport After ACL Reconstruction: A Systematic Review. Knee Surg Sports Traumatol Arthrosc. 2022;30(4):1369–79. 10.1007/s00167-021-06594-9. 33978778

[33] Nedder VJ, Raju AG, Moyal AJ, Calcei JG, Voos JE. Impact of Psychological Factors on Rehabilitation After Anterior Cruciate Ligament Reconstruction: A Systematic Review. Sports Health. 2025;17(2):291–8. 10.1177/19417381241256930. PMC11569572 39041333

[34] McPherson AL, Feller JA, Hewett TE, Webster KE. Psychological Readiness to Return to Sport Is Associated With Second Anterior Cruciate Ligament Injuries. Am J Sports Med. 2019;47(4):857–62. 10.1177/0363546518825258. 30753794

[35] Ueda Y, Matsushita T, Shibata Y, Takiguchi K, Ono K, Kida A, et al. Association Between Psychological Readiness to Return to Sports at 3 Months Postoperatively and Risk of Second ACL Injury. Orthop J Sports Med. 2024;12(4):23259671241239325. 10.1177/23259671241239325. PMC10996360 38584989

[36] Sell TC, Zerega R, King V, Reiter CR, Wrona H, Bullock GS, et al. Anterior Cruciate Ligament Return to Sport After Injury Scale (ACL-RSI) Scores Over Time After Anterior Cruciate Ligament Reconstruction: A Systematic Review With Meta-analysis. Sports Med - Open. 2024;10(1):49. 10.1186/s40798-024-00712-w. PMC11061071 38689130

[37] Lo CS, Chen KC, Shih JC, Cheng B, Chao WC. Integrating Psychological and Physical Assessments to Obtain Optimal Return-to-Sport Outcomes After ACL Reconstruction: A Systematic Review and Network Meta-analysis. J Sports Sci. 2025;43(20):2404–17. 10.1080/02640414.2025.2534224. 40665533

